# Regulation of Connective Tissue Growth Factor and Cardiac Fibrosis by an SRF/MicroRNA-133a Axis

**DOI:** 10.1371/journal.pone.0139858

**Published:** 2015-10-06

**Authors:** Aude Angelini, Zhenlin Li, Mathias Mericskay, Jean-François Decaux

**Affiliations:** 1 Biology of Adaptation and Ageing, Institut de Biologie Paris Seine (IBPS), DHU FAST Sorbonne Universités, UPMC Université Paris 06, Paris, France; 2 CNRS, UMR8256, Paris, France; 3 INSERM, U1164, Paris, France; Inserm, FRANCE

## Abstract

Myocardial fibrosis contributes to the remodeling of heart and the loss of cardiac function leading to heart failure. SRF is a transcription factor implicated in the regulation of a large variety of genes involved in cardiac structure and function. To investigate the impact of an SRF overexpression in heart, we developed a new cardiac-specific and tamoxifen-inducible SRF overexpression mouse model by the Cre/loxP strategy. Here, we report that a high level overexpression of SRF leads to severe modifications of cardiac cytoarchitecture affecting the balance between cardiomyocytes and cardiac fibroblasts and also a profound alteration of cardiac gene expression program. The drastic development of fibrosis was characterized by intense sirius red staining and associated with an increased expression of genes encoding extracellular matrix proteins such as fibronectin, procollagen type 1α1 and type 3α1 and especially connective tissue growth factor (CTGF). Furthermore miR-133a, one of the most predominant cardiac miRNAs, is strongly downregulated when SRF is overexpressed. By comparison a low level overexpression of SRF has minor impact on these different processes. Investigation with miR-133a, antimiR-133a and AdSRF-VP16 experiments in H9c2 cardiac cells demonstrated that: 1)–miR-133a acts as a repressor of SRF and CTGF expression; 2)–a simultaneous overexpression of SRF by AdSRF-VP16 and inhibition of miR-133a by a specific antimiR increase CTGF expression; 3)–miR-133a overexpression can block the upregulation of CTGF induced by AdSRF-VP16. Taken together, these findings reveal a key role of the SRF/CTGF/miR-133a axis in the regulation of cardiac fibrosis.

## Introduction

Heart failure (HF) remains a complex disorder that is characterized by substantial differences in levels of disease severity and progression. The process of left ventricular remodeling is an important indicator of morbidity and mortality in patients. Remodeling is characterized by changes in the geometry of the heart and, this process is essential to allow the adaptations of mechanical, chemical and electrical signals [1–3]. Because the cell-cell communication in cardiac remodeling is crucial, the most predominant changes affect the morphology of the cardiomyocytes, the development of myocardial fibrosis (fibroblast proliferation) and the amount and the organization of extracellular matrix (ECM) components [4–6]. Connective tissue growth factor (CTGF), a downstream mediator of the TGFß pathway, plays a major role in this adverse remodeling through the promotion of fibroblast proliferation and ECM production in connective tissues [7, 8].

Serum response factor (SRF), a MADS-box transcription factor, is essential for cardiac differentiation and maturation [9–12]. SRF binds to CArG-box DNA regulatory element, thereby regulating numerous genes involved in cell growth, migration, differentiation, cytoskeleton organization and energy metabolism. Previously we developed a mouse model of dilated cardiomyopathy (DCM) based on SRF gene disruption [13]. Triggering SRF loss in cardiomyocytes of adult mice led to a downregulation of proteins involved in contraction and energy transfer, a change in the architecture of the cardiac cells and a development of fibrosis [13–15]. SRF also regulates cardiac microRNAs such as miR–1 that we showed to repress NCX1 exchanger expression level in cardiomyocytes [16]. This leads to the development of DCM and all mice die from HF around 8–10 weeks after triggering SRF loss [13, 15]. These data showed a crucial role of SRF in the program of cardiac gene expression.

In contrast, few investigations have been done to better understand the consequences of a gradual SRF overexpression in heart. Previously, it has been reported that SRF mRNA level increases around 16% in the hearts of mice during aging [17]. In this present study, we analyzed the impact of two levels (low and high) of SRF protein overexpression in adult mouse cardiomyocytes on the development of cardiac fibrosis. We therefore created a new mouse model, involving cardiac-specific and tamoxifen-inducible SRF overexpression by the Cre/loxP strategy. Our findings demonstrate that a two-fold increase of SRF protein level has minor effect on cardiac structure and functions. When this level is higher than 2, the cardiomyocyte architecture was affected and the cardiac gene expression program disrupted leading to a rapid development of fibrosis. These findings reveal a key role of SRF, CTGF and miR-133a in the regulation of cardiac fibrosis leading progressively to HF.

## Materials and Methods

### Transgenic mice

All animal experiments must have been conducted according to the Ministere de l’Enseignement Superieur et de la Recherche, approved by the veterinary department of Paris to the legislation of R214-87 to R214-122 and R215-10 articles with the authorization number 75–835. All experimental procedures were done in accordance with institutional guidelines for animal experimentation. Mice were sacrificed by cervical dislocation.

**Fig 1:**
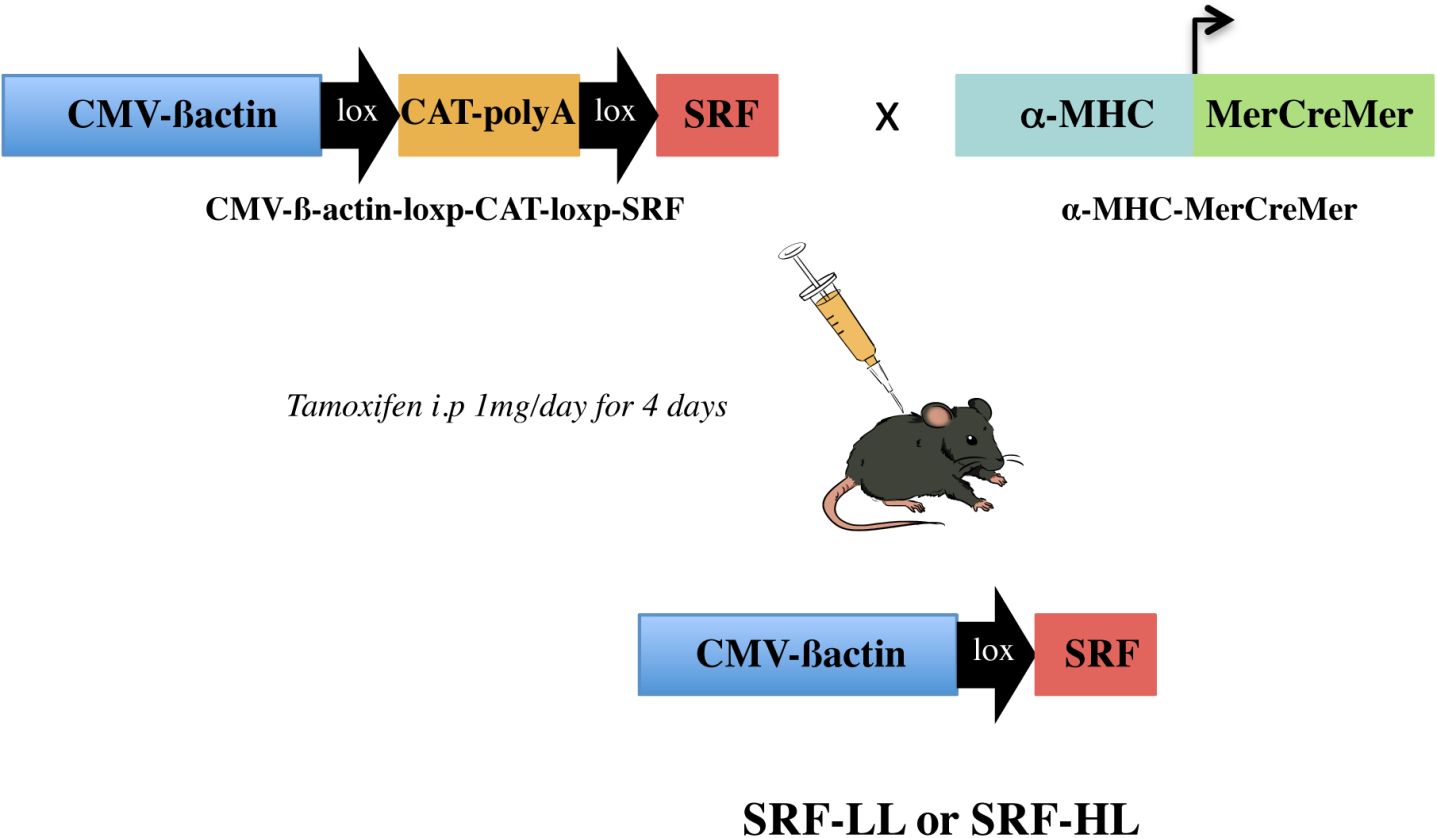
Inducible cardiac-specific SRF overexpression in adult mice. Schematic representation of the genetic strategy used to obtain a cardiac-specific SRF overexpression in adult mice. Tamoxifen injections to the double transgenic mice (α-MHC-MerCreMer/CAG-flCAT-SRF) induce excision of the floxed CAT gene that allows the expression of SRF gene.

Inducible SRF overexpression mice were generated using a transgene CAG-flCAT-SRF in which a mouse SRF cDNA is placed downstream to a floxed cassette containing a chloramphenicol acetyl transferase (CAT) reporter gene and a polyadenylation signal sequence that terminates transcription. The expression is under the control of the ubiquitous composite promoter made of cytomegalovirus immediate early enhancer and the chicken ß-actin promoter. It is only in the presence of Cre which excises the chloramphenicol acetyl transferase-PolyA sequence that SRF can be expressed. CAG-flCAT-SRF transgenic mice were crossed with the α-MHC-MerCreMer mice to generate α-MHC-MerCreMer/CAG-flCAT-SRF double transgenic mice (Fig 1). We obtained five lines of transgenic mice and selected two of them named SRF-LL (low level) and SRF-HL (high level) for detailed study. To generate α-MHC-specific overexpression, 2-month-old α-MHC-MerCreMer/CAG-flCAT-SRF were injected IP with 1 mg of tamoxifen (Sigma) in 50 μL of peanut oil for 4 days. Control mice were treated in the same conditions. All experiments were performed in mice 60 days after tamoxifen injections.

### H9c2 cell culture, infections and miR transfections

H9c2 rat cells (American Type Culture Collection Manassas, VA) were maintained in 100-mm dishes in standard Dulbecco’s modified Eagle’s medium (DMEM) supplemented with 10% fetal bovine serum (Sigma) and 100 U/mL penicillin/streptomycin at 37°C in a humidified atmosphere of 5% CO_2_. The day before the experiment, cells were plated in 6-well or 12-well plates at 1.5 10^5^ and 7.5 10^4^ respectively. They were grown in antibiotic-free fresh DMEM containing 10% fetal bovine serum. For infection experiments, cells were transduced with 2 i.p./cell of adenoviruses AdGFP or AdSRFVP16 for 8 hours, then fresh medium was added. We performed miR transfections using Lipofectamine RNAiMax (Invitrogen, France).

### ChIPs

H9c2 cells (5.10^6^) were fixed with 1% paraformaldehyde for 10 min. For all immunoprecipitations, sonicated chromatin was incubated with 3 μg anti-SRF (G20X, Santa Cruz) or IgG and immunocomplexes were recovered using a Magna ChIP G kit (Millipore). Quantification of immunoprecipitated DNA was done by real-time PCR using the appropriated primers and reported to input chromatin. Results shown are normalized to the recovery of a non-SRF-regulated gene region (in the intronic part of IL4).

### Histological analysis, immunohistochemistry and morphometric measurements

Histological analysis was performed by hematoxylin-eosin staining of paraffin-embedded heart sections. Fibrosis was detected by Sirius red (0.3%) staining for 1 hour followed by rapid acetic acid washing and coverslips were sealed in Eukitt mounting medium. Immunofluorescence analysis of frozen sections involved the following primary antibodies: anti-SRF (1: 500, Santa Cruz), anti-vinculin (1: 500, hVIN–1, Sigma), anti-CTGF (1: 200, Abcam), anti-vimentin (1:100, Progen) and anti-Ki67 (1: 750, Abcam) followed by incubation with Cy3- or Alexa488-coupled secondary antibody. Alexa Fluor 488-phalloidin (0.33 μmol/L, Sigma) was used to detect polymerized actin.

The maximal length and width of cardiomyocytes were measured by confocal microscopy following vinculin/phalloidin immunofluorescence staining using ImageJ software (version 1.39u). Ten to 15 fields corresponding to more than 150 cardiac cells per group of mice were analyzed. Measurements were performed on samples from at least three individuals in each group.

### Western blot analysis

Western blotting was performed as previously described [11] using anti-SRF (1: 800, Santa Cruz), anti-CTGF (1: 200, Abcam), anti-vimentin (1: 200, Progen) and anti-GAPDH (1: 2000, Santa Cruz) antibodies in 5% milk.

### Quantitative RT-PCR analysis

Total RNAs were extracted from hearts using TRI Reagent (Sigma) and were reversed transcribed with Moloney murine leukemia virus reverse transcriptase (Invitrogen) and random hexamers (Promega) to generate cDNAs. Total miRNAs were reversed transcribed using the miScript II RT Kit (Qiagen). PCR analysis was then performed with SYBR green PCR technology (Roche). The Primer3 program (frodo.wi.mit.edu/primer3/) was used to select primers (available on request). Cyclophilin mRNA was used as the reference transcript. For miRs, normalization was performed using miR–16 as reference.

### Statistical analysis

The significance of differences between means was assessed by Mann-Whitney test for non-gaussian data. P values of <0.05 were considered statistically significant.

## Results

### Cardiac cytoarchitecture is progressively modified by SRF overexpression

**Fig 2:**
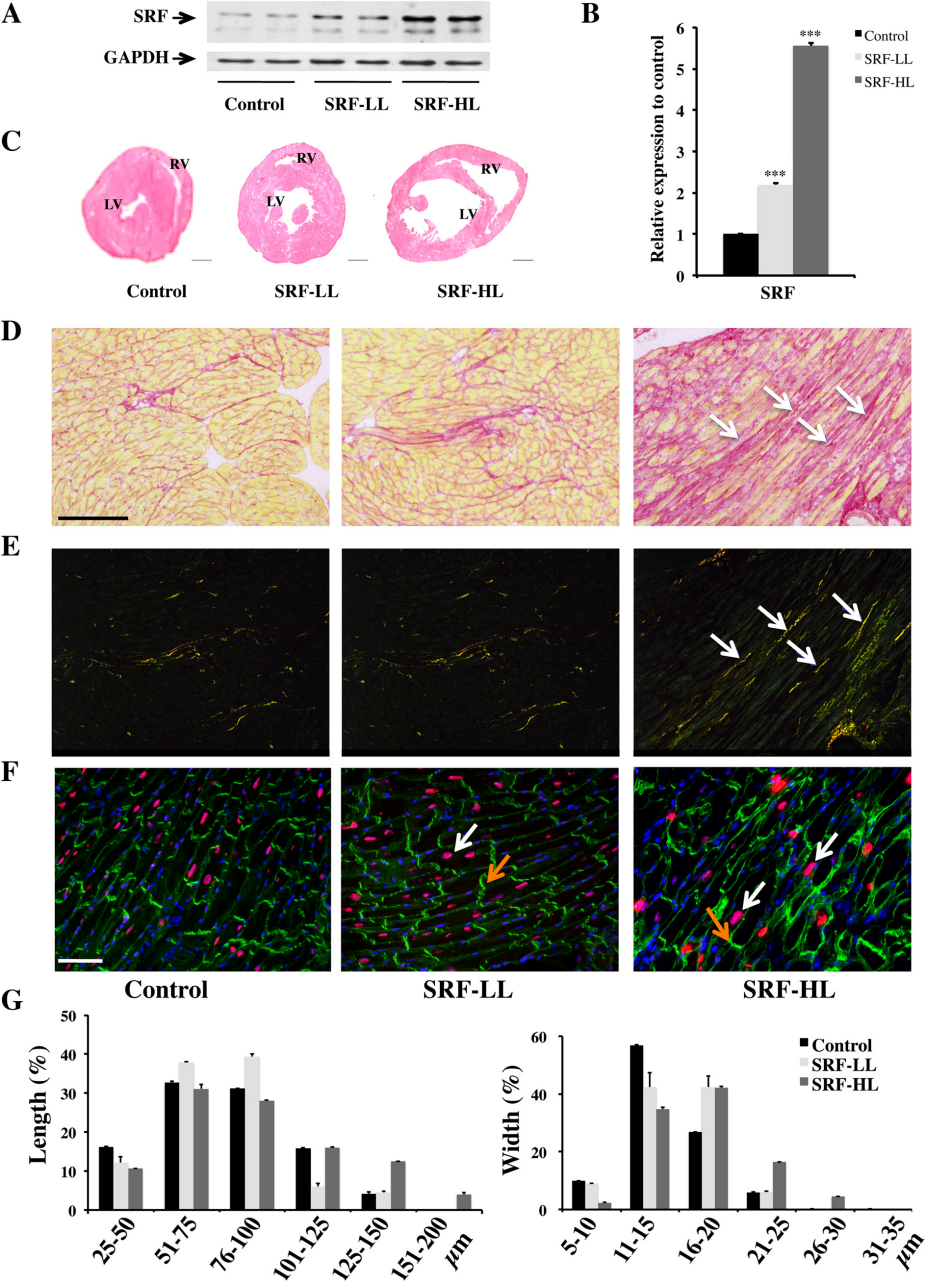
Effects of cardiac-specific overexpression of SRF on heart dilation and cardiomyocyte architecture. (A) Western blot analysis of SRF protein (10 μg of total protein per lane). The blot was also probed for glyceraldehyde-3-phosphate dehydrogenase (GAPDH) as a loading control. (B) Quantification of SRF protein level (n = 4). Data are presented as means ± s.e.m. *** indicates significant difference at P < 0.001, respectively versus the control group. (C) Hematoxylin-eosin staining of paraffin-embedded heart sections. The dilation of the ventricles is less marked in SRF-LL than in SRF-HL samples. LV: left ventricle; RV: right ventricle. These data are representative of three independent experiments. Scale bar: 1 mm. (D) Sections of mouse hearts were stained with Sirius red. The presence of endomyocardial fibrotic regions was observed in SRF-HL mice (white arrows). These data are representative of three independent experiments. Scale bar: 100 μm. (E) Identical fields than in D visualized with polarized light. Collagen fibers are highly birefrigent with fine fibers appearing green, and thicker fibers appearing yellow or orange (white arrows). (F) Confocal microscopy of cardiac sections labeled with anti-SRF antibody (red), anti-vinculin FITC (green) for cardiomyocyte membranes and DAPI (blue) for nuclei. SRF staining (white arrows) showing higher labeled cardiomyocyte nuclei in the SRF-HL than in the SRF-LL and in the control groups corroborating overexpression of the SRF gene. In the same way, intercalated discs (orange arrows) are substantially enlarged and irregularly shaped in the SRF-HL group compared with the SRF-LL and the control groups. These results are representative of three independent experiments. Scale bar: 80 μm. (G) Distribution of cardiomyocyte lengths and widths in the three groups of mice; n = 180 for each group (three different mice per group). SRF-LL: low level of SRF; SRF-HL: high level of SRF.

We generated five Cre-inducible transgenic lines allowing cardiomyocyte cell-specific SRF overexpression, we selected two of them with low and high SRF expression level for further studies (Fig 1). We analyzed three experimental groups of adult mice after tamoxifen injections: controls, a low level of SRF overexpression named SRF-LL and a high level of SRF overexpression named SRF-HL. By Western blot analysis, a 2 fold-increased of SRF protein is observed in the SRF-LL mice whereas a 5.5 fold increased is detected in the SRF-HL mice compared with the control mice (Fig 2A and 2B). Hearts of control and transgenic mice were harvested in diastole after 3 M KCl injection. Hematoxylin-eosin staining on heart sections shows a mild dilation of heart of the SRF-LL mice at 2 months after tamoxifen injections whereas a severe heart dilation of the SRF-HL mice was clearly observed at the same stage (Fig 2C). These results were confirmed by evaluation of the HW/BW ratio in the three groups of mice (S1 Fig). This ratio is significantly increased by 50% in the SRF-HL mice while it remains equivalent to the control in the SRF-LL mice (S1 Fig). SRF-LL mice were viable up to 12 months after tamoxifen injections with no sign of further dilation whereas all SRF-HL mice died within 10 weeks after tamoxifen injections. In the latter, hematoxylin-eosin staining revealed enlarged interstitial spaces between cardiomyocytes (S2 Fig). A different profile was observed in SRF-LL mice where interstitial spaces were smaller between cardiomyocytes and comparable to control mice (S2 Fig). The level of cardiac fibrosis was determined by Sirius red stained sections and light microscopy observations. We found that intense cardiac fibrosis was present in the heart of SRF-HL mice which expressed a high level of SRF (Fig 2D). In constrast, no fibrosis was observed in the heart of SRF-LL mice that were comparable to control group (Fig 2D). Observing identical field than in panel D with polarized light, we easily detected collagen fibers in the heart of SRF-HL mice. They are highly birefrigent with fine fibers appearing green, and thicker fibers appearing yellow or orange (Fig 2E). These structures were much less abundant in control and SRF-LL mice. Then we analyzed the changes in the cytoarchitecture associated with SRF overexpression in cardiomyocytes by confocal microscopic analysis. Heart sections were stained for SRF and for vinculin, a membrane associated cytoskeletal protein. A similar cytoarchitecture was observed in heart sections between SRF-LL and control mouse groups (Fig 2F). Their shape and alignment were homogeneous in size without gaps and, intercalated discs were thin and regular suggesting a correct force generation and transmission between these cells (Fig 2F). By contrast, a high increase of nuclear SRF protein disrupted cardiac cell architecture (Fig 2F). Cardiomyocytes were heterogeneous in size and were often stretched with enlarged interstitial spaces between them. Moreover, most of the intercalated discs were dysmorphic and thick (Fig 2F). Morphometric analysis revealed that SRF-HL cardiomyocytes were hypertrophied with increased length and width compared with SRF-LL and controls (Fig 2G). SRF-LL cardiomyocyte size distribution was closer to that of controls (Fig 2G).

### SRF overexpression altered cardiac gene expression program

**Fig 3:**
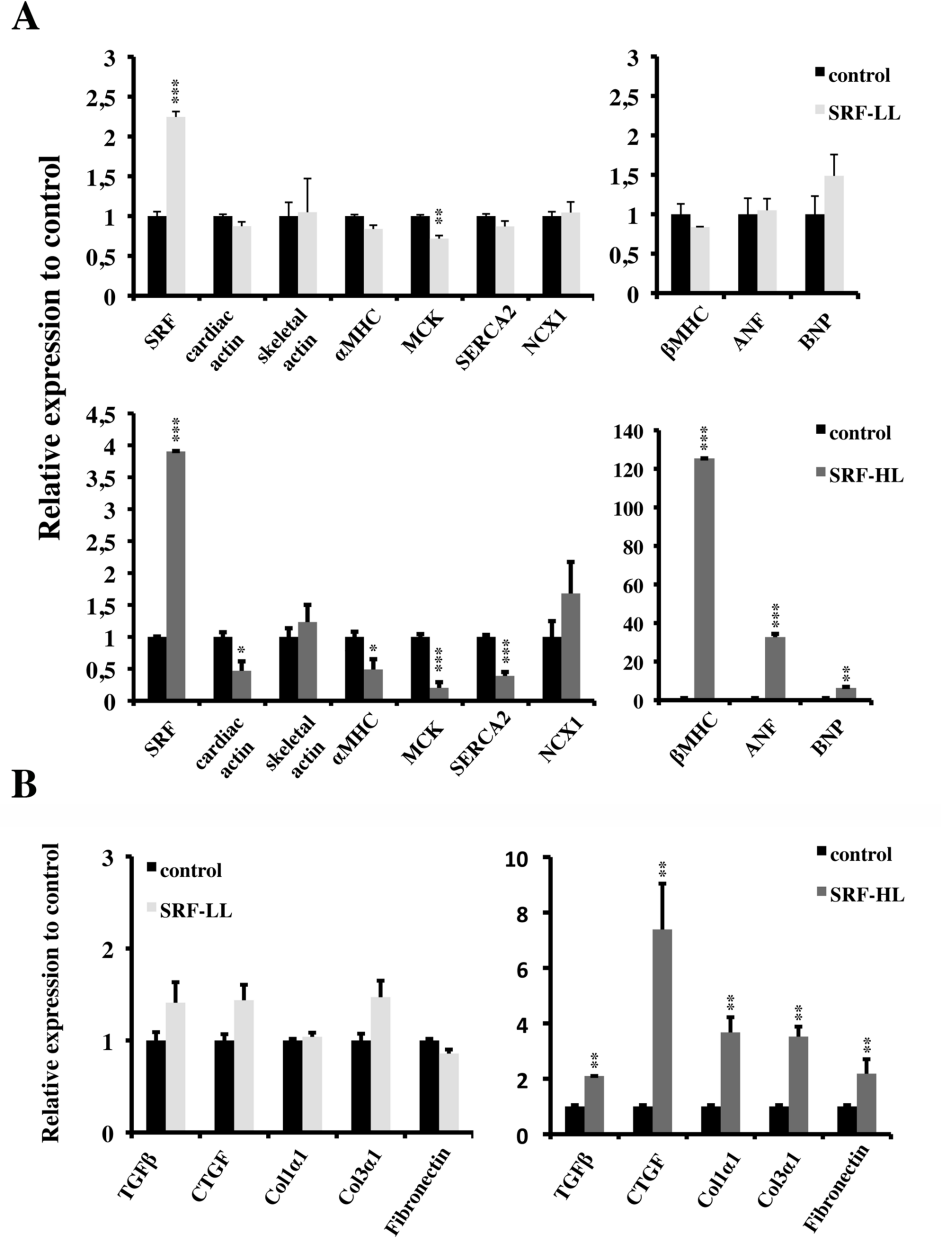
Impact of cardiac-specific overexpression of SRF on gene expression. qRT-PCR analysis of control (n = 7), SRF-LL (n = 6) and SRF-HL (n = 6) mouse mRNA, using cyclophilin as internal control. Data are presented as means ± s.e.m. *, ** and *** indicate significant difference at P < 0.05, P < 0.01 and P < 0.001, respectively versus the control group. (A) SRF target genes. (B) Genes involved in cardiac fibrosis. SRF-LL: low level of SRF; SRF-HL: high level of SRF.

We assessed the consequences of two levels of overexpression of SRF on cardiac gene expression by real-time RT-PCR. No specific disruption of the cardiac gene expression that we examined was observed by a 2-fold increase of SRF mRNA level in spite of a weak decrease of αMHC and MCK mRNA levels (Fig 3A). In contrast, a 4-fold increase of SRF mRNA level results in profound alterations of SRF target genes expression (Fig 3A). We noticed an important loss of cardiac α-actin mRNA level but no compensatory increase of skeletal α-actin mRNA level, the embryonic isoform (Fig 3A). Expression of two other SRF target genes, MCK involved in energy flux, and SERCA2 gene, encoding the sarcoplasmic reticulum Ca^2+^ pump ATPase was also decreased (Fig 3A). The myosin heavy chain (MHC) isoforms underwent a switch with a loss of the postnatal αMHC isoform, which is regulated by SRF and a strong increase of the embryonic βMHC isoform (Fig 3A). On the contrary to other SRF targets genes, the expression of two genes also described as SRF targets were maintained or increased including the Na^+^/Ca^2+^ exchanger NCX1 and the profibrotic gene CTGF (Fig 3B). Elevated BNP expression was also noticed in SRF-HL group of mice (Fig 3A).

To investigate the impact of SRF overexpression on the development of cardiac fibrosis, we quantified the expression of several genes involved in this process by RT-PCR. A dramatic increase of the expression of five tested genes was observed in SRF-HL group (Fig 3B). High level of TGFβ and CTGF mRNAs was detected (Fig 3B). Induction of CTGF by TGFβ has been documented in cardiac myocytes and fibroblasts. We also quantified the expression of genes encoding three ECM proteins secreted by cardiac fibroblasts, fibronectin, procollagen type 1α1 and type 3α1. As we expected, an important induction of these genes was shown in SRF-HL group (Fig 3B). In contrast, no significant modification of cardiac fibrotic gene expression program was detected in SRF-LL group (Fig 3B).

### SRF overexpression promotes cardiac fibrosis development by inducing CTGF expression in cardiomyocytes

**Fig 4:**
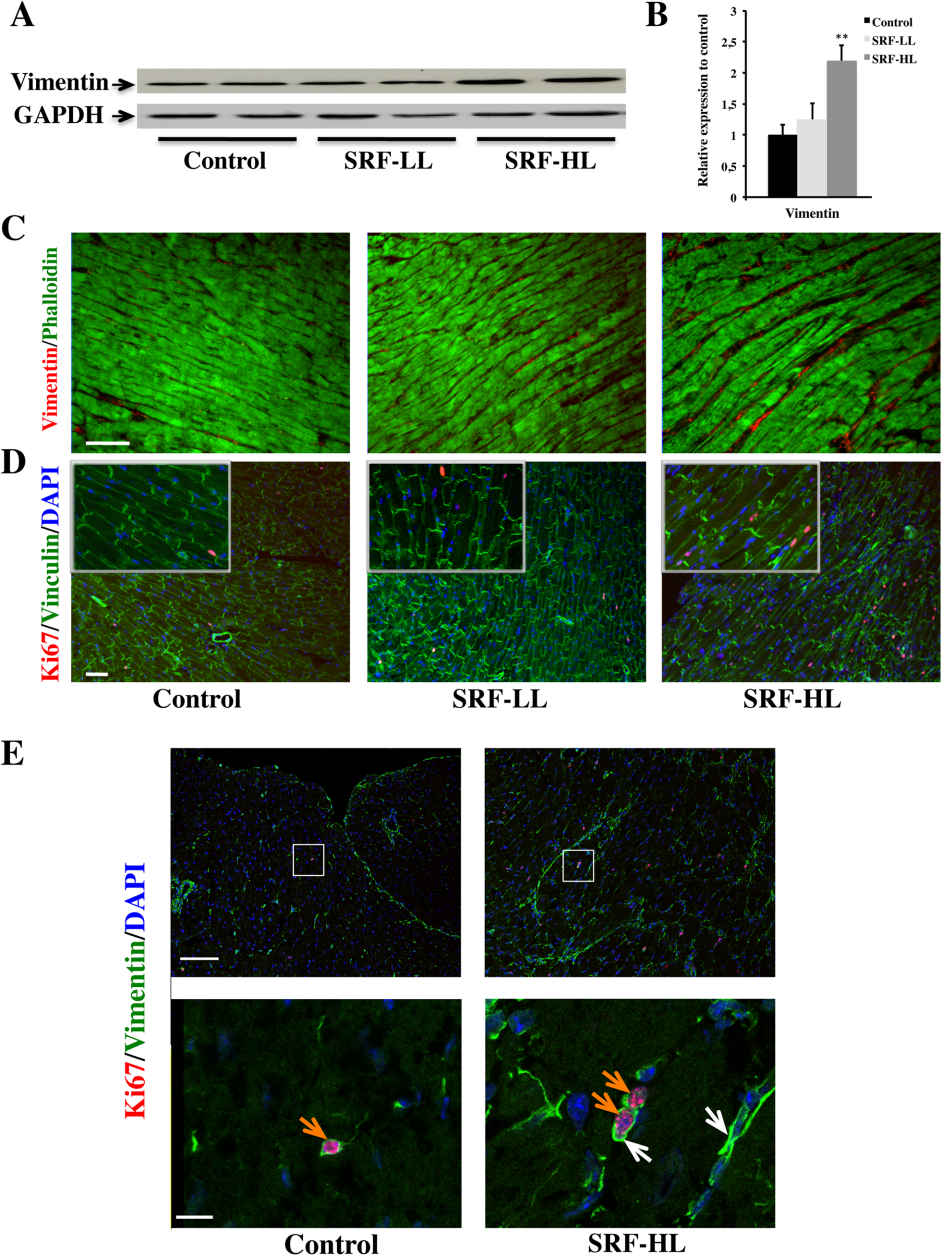
Impact of the level of SRF overexpression on cardiac fibrosis development. (A) Western blot analysis of vimentin protein. (B) Quantification of vimentin protein level (n = 4). Data are presented as means ± s.e.m. ** indicates significant difference at P < 0.01, respectively versus the control group. (C) Vizualisation of cardiac fibroblasts by vimentin staining of heart sections from control, SRF-LL and SRF-HL mice. Longitudinal frozen section showing the localization of cardiac fibroblasts stained with vimentin (red) and polymerized actin in myocytes labeled with Alexa-Fluor-488-phalloidin (green). These data are representative of three independent experiments. Scale bar: 80 μm. (D) Confocal microscopic view of Ki67 labeling (pink), nuclei (blue) and vinculin (green). Ki67 labeling is stronger in the SRF-HL than the SRF-LL and control mice and is exclusively located in the interstitial cells. The enlargement is of twice. These data are representative of three independent experiments. Scale bar: 80 μm. (E) Confocal microscopic view of Ki67 labeling (pink), vimentin (green) and nuclei (blue) of heart sections from SRF-HL and control mice. Top panel, scale bar: 100 μm; bottom panel corresponding to the square indicated in the top panel, scale bar: 10 μm. These data are representative of two independent experiments.

The effect of SRF overexpression on the expression of vimentin, a marker of cardiac fibroblasts, was assessed in the heart by immunoblotting and immunostaining. A 2-fold increase of vimentin protein level was showed in SRF-HL compared with control and SRF-LL groups (Fig 4A and 4B). Vimentin and phalloidin co-immunostaining revealed overgrowing cardiac fibroblasts within the large interstitial spaces observed in SRF-HL heart but not in control and SRF-LL hearts (Fig 4C). Co-immunostaining for Ki67 and vinculin showed that the cardiomyocytes were not replicating but that many interstitial cells, mainly cardiac fibroblasts, were dividing in SRF-HL mice than in control and SRF-LL groups (Fig 4D). Quantification confirmed that 4 times more Ki67-positive per heart sections were present in SRF-HL mice than in control and SRF-LL groups (data not shown). Then we performed a co-immunostaining for Ki67 and vimentin (Fig 4E). The vast majority of Ki67-positive interstitial cells were also vimentin-positive showing that cardiac fibroblasts are proliferating when SRF is strongly expressed in cardiomyocytes (Fig 4E, bottom, magnification of the white square field).

**Fig 5:**
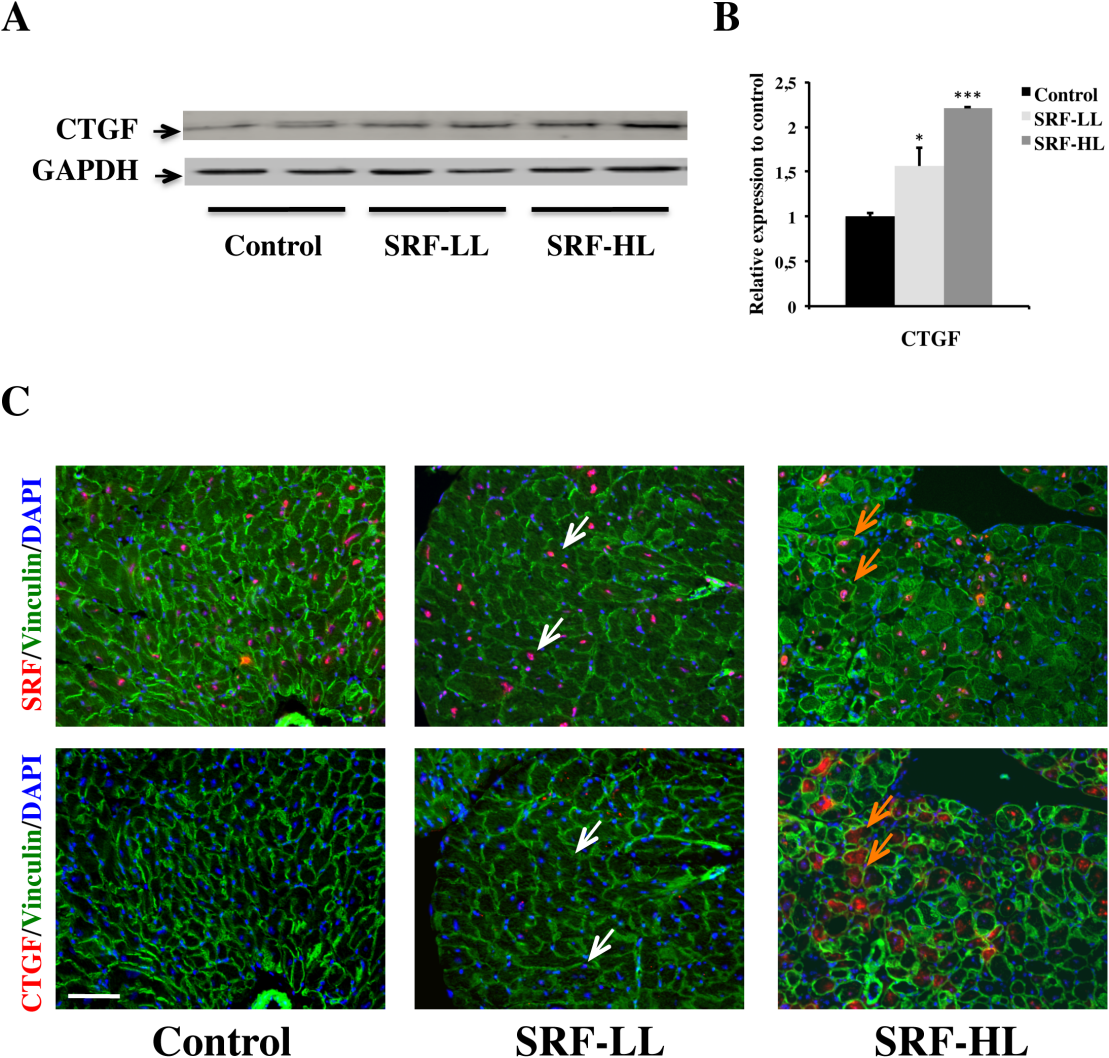
Cardiac CTGF expression induced by a high SRF overexpression. (A) Western blot analysis of CTGF protein. The GAPDH blot represented here is the same used for vimentin in Fig 4 since CTGF and vimentin were blot on the same membrane. (B) Quantification of CTGF protein level (n = 4). Data are presented as means ± s.e.m. * and *** indicate significant difference at P < 0.05 and P < 0.001, respectively versus the control group. (C) Immunoflurescence labeling of SRF (red), vinculin (green) and nuclei (blue) (upper line), and CTGF (orange), vinculin (green) and nuclei (blue) (lower line) on serial heart sections of control, SRF-LL and SRF-HL mice. The white arrows indicate SRF-positive/CTGF-null cardiomyocytes while the orange arrows indicate SRF-positive/CTGF-positive cardiomyocytes. All these data presented in this figure are representative of three independent experiments. Scale bar: 80 μm.

The effect of overexpression of SRF on the expression of CTGF, a secreted ECM-associated protein, was assessed in the heart by immunoblotting and immunostaining. A 1.5-fold and a 2.2-fold increase of CTGF protein level was observed in SRF-LL and SRF-HL respectively compared with control group (Fig 5A and 5B). Then we performed immunohistochemical detection on serial heart sections to determine the cell specificity of its protein expression pattern. In control and SRF-LL hearts, CTGF immunoreactivity was practically undetectable (Fig 5C). By contrast, in SRF-HL hearts where SRF is strongly expressed, CTGF was mainly abundant in cytoplasm of cardiomyocytes and to a lesser extent in interstitial spaces corresponding to proliferating fibroblasts (Fig 5C). Taken together, these findings suggest that a massive increase of SRF expression leads to CTGF overexpression in cardiomyocytes inducing proliferation and CTGF expression in cardiac fibroblasts.

### SRF and miR-133a modulate CTGF gene expression

**Fig 6:**
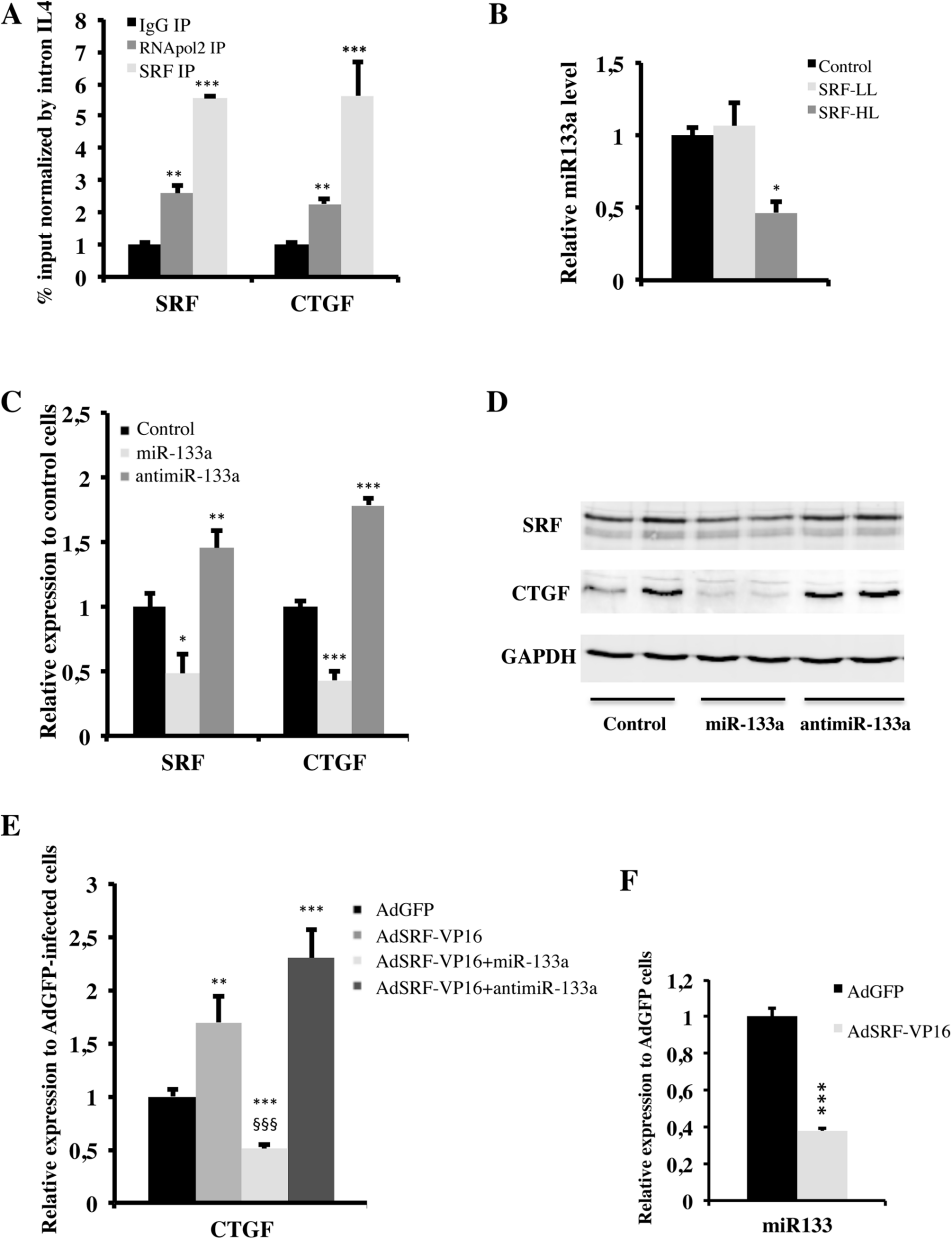
Involvement of SRF and of miR-133a in CTGF regulation. (A) ChIP was performed using H9c2 cells and antibodies specific to SRF, RNApol2 and IgG. Bound SRF or CTGF promoters was amplified by qRT-PCR and normalized to input and to an additional negative control (primers spanning the first intron of Il4). Data are mean± s.e.m. ** and *** indicate significant difference at P < 0.01 and P < 0.001, respectively versus IgG. Data are representative of 4 independent experiments. (B) qRT-PCR assays of miR-133a in cardiac tissue from control (n = 7), SRF-LL (n = 6) and SRF-HL (n = 6) mouse miRNA, using miR–16 as internal control. Data are presented as means ± s.e.m. * indicates significant difference at P < 0.05, respectively versus the control group. (C) H9c2 cells were not treated (n = 7) or treated by miR-133a (50 nM) (n = 7) or antimiR-133a (50 nM) (n = 7) for 48 hours then scratched and total RNAs were extracted. Data are presented as means ± s.e.m. *, ** and *** indicate significant difference at P < 0.05, P < 0.01 and P < 0.001, respectively versus the control group. (D) Western blot analysis of SRF, CTGF and GAPDH proteins (20 μg of total protein per lane). This blot is representative of three independent experiments. (E) H9c2 cells were transduced with AdGFP (n = 5) or AdSRF-VP16 (n = 5) adenoviruses for 8 hours, the medium was changed and treated or not by miR-133a (50 nM) or antimiR-133a (50 nM). 48 hours later, cells were scratched and total RNAs extraction was done. Data are presented as means ± s.e.m. ** and *** indicate significant difference at P < 0.01 and P < 0.001, respectively versus AdGFP; §§§ indicates significant difference at P < 0.001, respectively versus AdSRF-VP16. (F) H9c2 cells were transduced with AdGFP (n = 5) or AdSRF-VP16 (n = 5) adenoviruses for 8 hours then fresh medium was added. 48 hours later, cells were scratched and total RNAs extraction was done. Data are presented as means ± s.e.m. *** indicates significant difference at P < 0.001, respectively versus AdGFP.

Previous studies indicated that a "CArG" box has been characterized in the CTGF regulatory region and located at position—3791 upstream of the transcription start site [18]. Moreover, the SRF gene contains two "CArG" boxes in its promoter and autoregulates its own expression [19]. We first showed by ChIP assays performed in H9c2 cardiac cells that SRF binds to its own "CArG" boxes used as positive control of this experiment. We demonstrated that the CTGF regulatory region covering position—3791 was robustly amplified from the SRF immunoprecipitate compared to IgG controls, confirming SRF binding (Fig 6A).

MiR-133a is regulated by SRF in skeletal myoblast cells [20] and has been involved in the repression of CTGF mRNA in fibroblast cell lines [21] but these regulations are not known in the heart. To assess the impact of SRF on miR-133a expression in the heart, we estimated the miR-133a levels in SRF-LL and SRF-HL hearts of mice. We showed a 50% decrease of miR-133a expression level in SRF-HL compared with control hearts, however this decrease was not seen in SRF-LL hearts (Fig 6B). These data indicate that only a high level of SRF in cardiomyocytes leads to a decrease of miR-133a level. To examine the respective roles of SRF and of miR-133a on CTGF gene expression, H9c2 cells derived from rat neonate cardiomyocytes were transfected with a miR-133a mimic for 48 hours (Fig 6C). Under these conditions, a significant downregulation (≈ 50%) of SRF and of CTGF mRNAs was observed in the cells treated with miR-133a compared with those treated with the control mimic (Fig 6C). By contrast, a 1.5 and a 1.8 fold increase in SRF and CTGF mRNA levels respectively were observed in the cells treated with the miR-133a inhibitor compared with the control group (Fig 6C). Western blot analysis revealed that, while miR-133a mimic treatment decreased SRF and CTGF protein expression, the opposite result was observed by following antimiR-133a treatment (Fig 6D). In cardiac H9c2 cells, we overexpressed a constitutively active SRF by AdSRF-VP16 that activates its target genes. Infection of H9c2 cells by AdSRF-VP16 led to an induction of CTGF mRNA level (1.8 fold) compared with cells infected by AdGFP (Fig 6E). MiR-133a mimic was able to block the induction of CTGF by AdSRF-VP16 and to decrease CTGF mRNA level 50% compared with the cells treated with the control mimic (P < 0.001). In contrast, transfection of the antimiR-133a in presence of AdSRF-VP16 further increased CTGF mRNA level (2.4 fold) (P < 0.001) (Fig 6E). In addition, we showed in AdSRF-VP16 cells that the increase of SRF expression induced a down-regulation of miR-133a expression by 60% compared with cells infected by AdGFP (Fig 6F).

## Discussion

Cardiac remodeling is defined by the process of left ventricle adaptation to different forms of physiopathological hits. Major features of cardiac remodeling include changes in tissue architecture such as hypertrophy of cardiac myocytes and myocardial fibrosis development. SRF is a transcription factor implicated in the regulation of a large variety of genes involved in cardiac structure and function. Numerous data established its key role in cell growth and differentiation. We previously reported that triggering SRF gene disruption in adult cardiomyocytes led to DCM characterized by LV dilation, a progressive loss in contractility and fatal HF [13]. Here, we report an analysis of the effects of cardiac-specific SRF overexpression on fibrogenic process. Our findings demonstrate that a high level of overexpressed SRF in adult mice heart induces a drastic fibrosis whereas a low level has no impact on this process. We showed that CTGF, a secreted factor associated with the ECM, and miR-133a are strongly involved in this mechanism of cardiac fibrosis. In the cardiomyocytes, the regulation of CTGF and miR-133a expression is directly linked to SRF protein level.

We developed a mouse model involving cardiac-specific and tamoxifen-inducible SRF overexpression by the Cre/LoxP strategy to estimate the consequences of a low level (SRF-LL) or a high level (SRF-HL) of SRF protein expression on cardiac fibrosis. We showed that an increase of 2 fold of SRF protein level has no dramatic effect on cardiac cytoarchitecture with only mild dilation of the ventricular chambers. The shape and the alignment of the cardiac cells were regular and the intercalated disks remained thin. By contrast, in SRF-HL transgenic mice when the amount of SRF protein is higher than 2, we observed a rapid dilation of the heart linked to a myofibrillar disorganization and a stretching of the intercalated disks involved in force transmission, which are characteristic features of DCM. In the same line, constitutive SRF overexpression starting during embryonic development led to severe cardiac dysfunction although the majority of transgenic founder mice were unable to generate progeny [22].

To evaluate the effects of SRF overexpression on cardiac gene expression, a panel of cardiac genes regulated or not by SRF was selected. Interestingly, the pattern of expression of SRF regulated genes was seriously affected only by a high SRF overexpression. Several of them were downregulated (cardiac α-actin, αMHC and MCK). These observations suggest a mechanism of “transcriptional squelching” that has already observed for SRF in other cellular contexts [23, 24]. In this type of dysregulation by “transcriptional squelching”, the overexpressed SRF can lead to a titration of one or several essential cofactors present in limiting amounts in cardiac cells. At high level of overexpression, all CArG boxes (SRF binding sites) are probably saturated with SRF dimers but any additional SRF not bound to DNA is competing with DNA-bound SRF for cofactor interaction that normally help to recruit RNA Pol2 at target genes. Indeed, SRF has been described as a transcription factor with low intrinsic transcriptional activity that works mainly by attracting to its target promoters other transcription factors such as GATA4, Nkx2.5 and transcriptional factors of different families, notably of the myocardin family (Myocardin, MRTF-A or MRTF-B), that possess potent transactivation domains [25–28]. Finally, a very high level of SRF overexpression affects the expression of a majority of SRF target genes and is functionally similar to SRF inactivation [13, 15]. There is however some exceptions among the SRF target genes. We observed a higher expression in SRF-HL hearts of NCX1 and CTGF gene that were also described as SRF target genes [18, 29, 30], suggesting a different mode of regulation for these genes by SRF.

Analysis of the mammalian ‘CArGome’ (CArG elements in the genome) identified CTGF as a potential SRF target and the presence of a “CArG” box located at position -3791 bp from the start site of transcription in the CTGF promoter [31], which has been validated in human umbilical vein endothelial cells [18]. In spite of the presence of one mismatch compared to the canonical CC(A/T)GG sequence [31], we found that SRF is able to bind to this “CArG-like” box in H9c2 cells, a cardiac cell line. Moreover, infection of these cells by adSRF-VP16 induces CTGF mRNA level indicating that SRF could control CTGF transcription by acting on the “CArG” box located in the regulatory region.

So the question arises: Why CTGF is not repressed by transcriptional squelching like the other SRF target genes? In the last few years, it has become clear that MiRNAs are dominant players in different aspects of cardiac remodeling, including fibrosis [32, 33]. Interestingly, it was previously shown that the CTGF transcript is a target of miR-133a [21]. MiR-133a is one of the most predominant expressed miRNAs in cardiac cells and it plays a crucial role in cardiac development and pathophysiology [20, 34–37]. MiR-133a is expressed under the control of MEF2 and SRF transcription factors in cardiac and skeletal muscle cells [38]. Reciprocally miR-133a targets SRF mRNA and limits its expression [38].

In the present study, we established that the level of miR-133a decreased in the heart of SRF-HL mice while it remained unchanged in the heart of SRF-LL mice suggesting that the level of miR133a as other SRF target genes is repressed by high SRF concentration in cardiomyocytes. Based on several experiments linked to an overexpression or an inhibition of miR-133a expression using a specific antimiR in cultured cardiac cells, we show that miR-133a acts on the regulation of SRF and CTGF expression as a repressor. These results are in agreement with previous findings showing that miR-133a represses SRF [36] and CTGF [21]. In these studies, direct binding of miR-133a to SRF and CTGF 3’UTR has already been demonstrated using luciferase assays [21, 36]. Here we show that AdSRF-VP16 is able to increase CTGF expression and to decrease miR-133a level simultaneously. Moreover, inhibiting miR-133a on top of SRF-VP16 overexpression further induced CTGF expression in H9c2 cells. In contrast, when miR-133a is upregulated, CTGF expression is decreased in spite of the overexpression of SRF by AdSRF-VP16 suggesting that miR-133a is a powerful repressor of CTGF expression. This regulatory mechanism in which a transcription factor (SRF) regulates both a coding gene (CTGF) and a microRNA (miR-133a) involved in the repression of the mRNA of this gene seems to be a characteristic feature of SRF mode of regulation on a subset of target genes whose expression should be limited at baseline in the heart. Indeed we have previously shown a similar regulatory loop between SRF, its target gene NCX1 and its target microRNA miR–1 [16].

Numerous reports have shown that cardiac fibrosis is an important contributor to the development of cardiac dysfunction in diverse pathological situations [39–41]. These studies underscore the important role played by TGFß and one of its downstream mediator CTGF [7, 8]. CTGF has been found elevated in almost any human disorder which exhibit excessive scarring and fibrosis including cardiac disease [42]. Consistent with these findings, levels of the transcripts of these two factors were closely correlated and higher in hearts of SRF-HL than SRF-LL and control mice. Many *in vitro* studies have shown that CTGF stimulates the proliferation of fibroblasts, their differentiation towards myofibroblasts and increases ECM production [43–48]. In heart of SRF-HL mice, vimentin, a marker of cardiac fibroblasts [49], procollagen type 1α1 and type 3α1 and fibronectin were also strongly increased suggesting an activation of the fibroblast proliferation. This was confirmed by an increase of the number of Ki67-positive cells found almost exclusively in vimentin-positive population. In the healthy heart, CTGF is mainly expressed in fibroblasts, by contrast cardiac remodeling also leads to CTGF secretion in cardiac cells [41, 42, 50]. In SRF-HL mice, we noticed an increase of CTGF expression in cardiomyocytes in addition to cardiac fibroblasts. CTGF was not detected in cardiac cells from SRF-LL mice expressing SRF at a low level. The abundance of CTGF-positive cardiomyocytes in SRF-HL hearts correlated with the degree of the interstitial fibrosis and the increase of fibronectin, procollagen type 1α1 and 3α1 expression suggesting that CTGF produced and secreted by the cardiomyocytes can exert a paracrine control on cardiac fibroblast proliferation.

We have previously shown that CTGF is also upregulated in the cardiomyocytes after SRF deletion in the adult heart (SRF-HKO) leading to DCM and cardiac fibrosis [15] in a process similar to the one observed in the SRF-HL transgenic mice. Although seemingly paradoxical, these results can be reconciled by the fact that, as explained previously, very high levels of SRF leading to transcriptional squelching are functionally comparable to SRF deletion. In agreement with this hypothesis we found that miR-133a is strongly downregulated in the SRF-HKO myocardium as in the SRF-HL mice (data not shown), reinforcing the concept that miR-133a is a downstream target of SRF and a major determinant of CTGF expression level.

**Fig 7:**
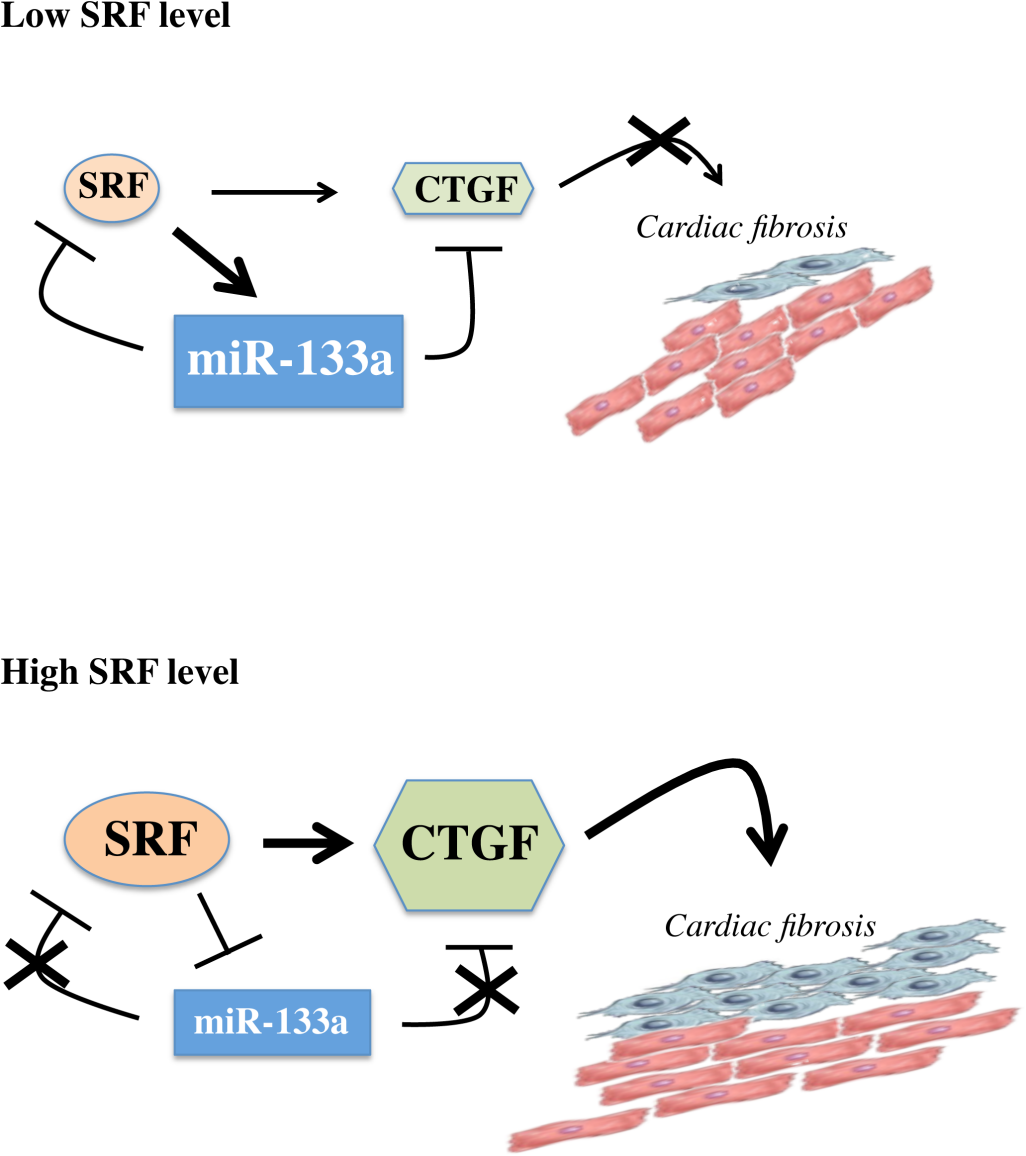
A scheme illustrating the loop of regulation between SRF, CTGF and miR-133a in low and high SRF expression level in cardiomyocytes.

In summary, our results reveal that SRF plays a role of homeostatic regulator between cardiomyocytes and fibroblasts in the heart and, too much SRF (this study) or not enough SRF [15] is deleterious for this balance. These data are relevant in a clinical context since overexpression of a SRF activator as been reported in human hearts with idiopathic cardiomyopathy [9] and conversely repression of SRF pathway is proposed to be a major pathogenic mechanism in several forms of DCM [51, 52]. A major point of this study demonstrates that a novel axis composed by SRF, CTGF and miR-133a is involved in the regulation of cardiac fibrosis. At low SRF level, miR-133a concentration is elevated by the action of SRF and provides a negative feedback loop on SRF and on CTGF allowing a steady state between cardiomyocyte and fibroblast populations (Fig 7). Conversely, a high level of SRF results in reduced expression of miR-133a followed by an elevation of the expression CTGF and a dramatic rise of fibrosis (Fig 7). Previous data have shown that miR–133 is downregulated in cardiac hypertrophy [34].

Our findings suggest that restoring miR–133 expression in the context of cardiac hypertrophy could help to repress the impact of SRF/CTGF axis on cardiac fibrosis.

## Supporting Information

S1 Fig Measurements of Heart Weight/Body Weight (HW/BW) in the three groups of mice. HW/BW (mg/g) in control (n = 7), in SRF-LL (n = 6) and in SRF-HL (n = 6) mice. (A) Histogram. (B) Table. Data are presented as means ± s.e.m. *** indicates significant difference at P < 0.001, respectively versus control mice.
(TIF)

S2 Fig Hematoxylin-eosin staining of paraffin-embedded sections. The presence of frequent intercellular gaps was observed in SRF-HL hearts. These data are representative of three independent experiments. Scale bar: 20 μm.
(TIF)
