# Supplementary figures and images for: Regulation of Connective Tissue Growth Factor and Cardiac Fibrosis by an SRF/MicroRNA-133a Axis
Source: PLoS One. 2015 Oct 6;10(10):e0139858. doi: 10.1371/journal.pone.0139858 (PMC4595333; doi:10.1371/journal.pone.0139858)

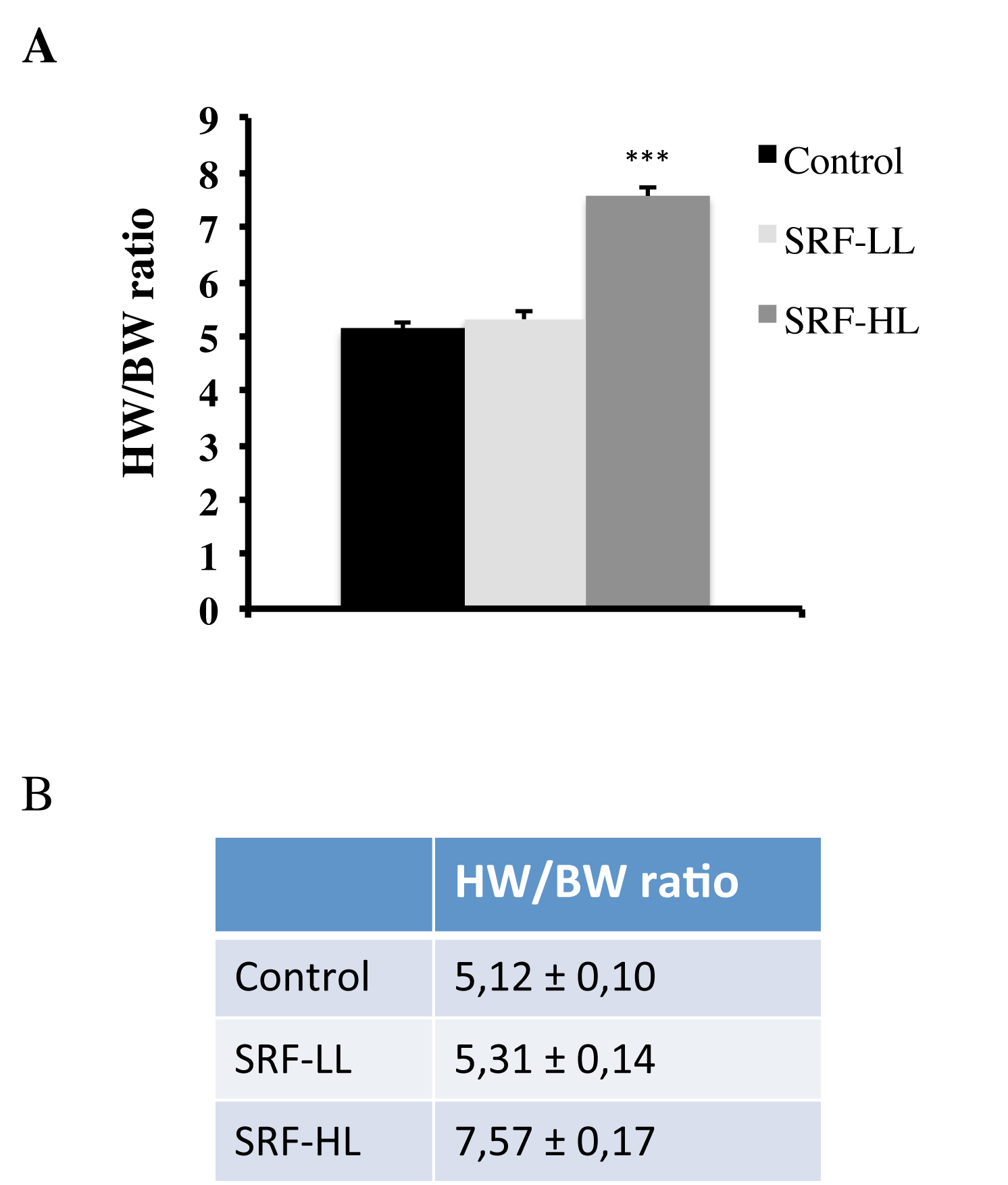

Supplement: S1 Fig — HW/BW (mg/g) in control (n = 7), in SRF-LL (n = 6) and in SRF-HL (n = 6) mice. (A) Histogram. (B) Table. Data are presented as means ± s.e.m. *** indicates significant difference at P < 0.001, respectively versus control mice. (TIF) [file pone.0139858.s001.tif]

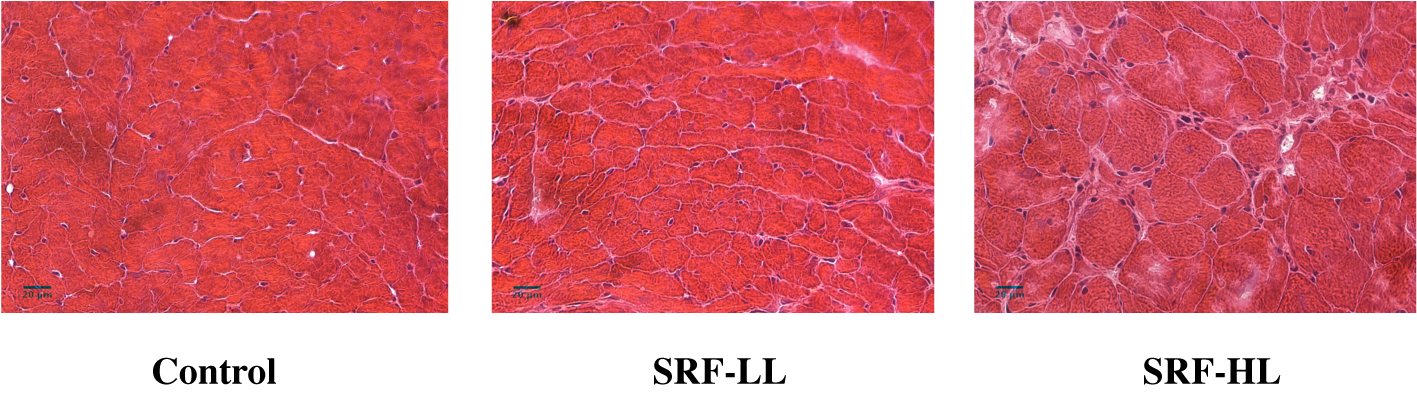

Supplement: S2 Fig — The presence of frequent intercellular gaps was observed in SRF-HL hearts. These data are representative of three independent experiments. Scale bar: 20 μm. (TIF) [file pone.0139858.s002.tif]
